# A phase 1 trial of iron metabolism-targeting oral gallium maltolate in recurrent and refractory glioblastoma

**DOI:** 10.1093/noajnl/vdag154

**Published:** 2026-06-08

**Authors:** Jennifer M Connelly, Casey J Zoss, Aniko Szabo, Shama P Mirza, Frederick Adom, Lawrence R Bernstein, Mona M Al-Gizawiy, Kathleen M Schmainda, Christopher R Chitambar

**Affiliations:** Department of Neurology, Medical College of Wisconsin, Milwaukee, Wisconsin, USA; Department of Biophysics, Medical College of Wisconsin, Milwaukee, Wisconsin, USA; Department of Biostatistics, Medical College of Wisconsin, Milwaukee, Wisconsin, USA; Department of Chemistry and Biochemistry, University of Wisconsin, Milwaukee, Wisconsin, USA; Department of Chemistry and Biochemistry, University of Wisconsin, Milwaukee, Wisconsin, USA; Gallixa LLC, Menlo Park, California, USA; Department of Biophysics, Medical College of Wisconsin, Milwaukee, Wisconsin, USA; Department of Biophysics, Medical College of Wisconsin, Milwaukee, Wisconsin, USA; Department of Medicine, Division of Hematology and Oncology, Medical College of Wisconsin, Milwaukee, Wisconsin, USA; Department of Biophysics, Medical College of Wisconsin, Milwaukee, Wisconsin, USA

**Keywords:** clinical trial, gallium maltolate, glioblastoma, iron targets, Phase 1

## Abstract

**Background:**

Gallium maltolate is an oral compound that disrupts iron-dependent glioblastoma (GBM) growth and prolongs survival in animal models. This Phase 1 trial of oral gallium maltolate in patients with recurrent GBM was conducted to assess toxicity, determine a recommended Phase 2 dose (RP2D), and seek signals of tumor response.

**Methods:**

Using a 3 + 3 dose-escalation design with 5 drug dose levels of oral gallium maltolate (500-2,500 mg daily), 24 patients received a minimum of one 4-week cycle to assess toxicity, and 22 of these patients received a minimum of 2 cycles to assess tumor response. Patients with stable disease on MRI at 8 weeks continued treatment until progression.

**Results:**

Grade 1 or 2 adverse effects of diarrhea, anorexia, nausea, and fatigue occurred in 24 patients. The RP2D was determined by considering diarrhea requiring treatment and serum gallium levels that plateaued at doses of 2,000-2,500 mg/day. ALT elevation occurred in 1 patient each on 1,000 and 2,000 mg/day. RBC MCV decreased below normal during treatment in 9 patients. Nephrotoxicity was not seen. GBM progressed in 11 of 22 evaluable patients after 2 cycles and in 6 following 3-6 cycles. Three patients progressed after 8, 10, and 26 cycles. One patient with a stable partial response remains on treatment after 33 cycles. The median overall survival was 16 months.

**Conclusions:**

Oral gallium maltolate is safe and well-tolerated with an RP2D of 2,000 mg/day. Further investigation to assess the clinical efficacy of gallium maltolate in GBM is warranted.

Key Points

- Novel metallodrug for glioblastoma
- Iron targeting mechanisms of action

Importance of the Study

Innovative drugs with novel mechanistic actions are sorely needed for the treatment of glioblastoma (GBM). Our preclinical research has identified gallium maltolate [tris(3-hydroxy-2-methyl-4-pyranato)-gallium] as a new compound that targets the iron-dependent growth of GBM stem cells and cell lines; it retards GBM tumor growth and prolongs survival in rats with orthotopically implanted human GBM xenografts. We conducted a Phase 1 dose-escalation clinical trial of oral gallium maltolate at daily dose levels of 500-2,500 mg in patients with recurrent or treatment-refractory GBM and showed that this drug is well tolerated and safe without renal or hepatic toxicity. Only grade-1 or -2 adverse effects were observed. This study is important because it is the first Phase 1 trial of this drug in GBM. It establishes a ­recommended Phase 2 dose for future trials and suggests signals of tumor response that warrant further evaluation.

The median survival of newly diagnosed patients with ­glioblastoma (GBM) treated with surgical resection, chemoradiation, and maintenance temozolomide (TMZ) is 14-16 months.^1^^,^^2^ The addition of tumor-treating fields (TTFields) in selected patients receiving maintenance TMZ extends the median ­survival to 20 months.^2^^,^^3^ Unfortunately, the majority of GBM patients develop a recurrence within a relatively short time following completion of initial treatment. A recent meta-analysis of treatment options for recurrent GBM ­conducted by McBain et al reported that the median progression-free survival (PFS) of these patients ranged from 1.5 to 4.2 months, while the median overall survival (OS) ranged from 5.5-12.6 months.^4^ Montemurro et al conducted a review of 17 studies comprising 219 patients with recurrent GBM treated with Laser Interstitial Thermal Therapy (LITT) and reported a median PFS and median OS of 5.6 and 10.2 months, respectively.^5^ Thus, there remains an urgent need to develop additional drugs for GBM with novel mechanisms of antineoplastic action that differ from existing therapeutic approaches.

One targetable pathway in GBM that has remained underexplored involves tumor iron homeostasis, which is known to be altered in malignant cells.^6^^,^^7^ Compared with normal cells, cancer cells require high amounts of iron to support iron-dependent processes altered in malignancy.^7^^,^^8^ Gallium is a group 13 metal that is trivalent (Ga^3+^) under physiologic conditions. Preclinical research has shown that due to the close chemical similarity between Ga^3+^ and ferric iron (Fe^3+^), gallium can bind the iron transporter transferrin (Tf) to form Tf-Ga which is taken up by cells via Tf receptor1 (TfR1).^9^^,^^10^ TfR1 is highly expressed in GBM cells, making them good targets for gallium-based therapeutics.^11-13^ Gallium exerts its antineoplastic activity by a 3-pronged attack on iron-driven processes altered in malignancy; these processes include TfR1-mediated cellular iron uptake, iron-dependent ribonucleotide reductase (RR), and iron-sulfur (Fe-S) clusters essential for the activity of complex 1 of the mitochondrial electron transport chain.^9^^,^^13-20^

Gallium has been shown to cross the blood-brain barrier (BBB) and enter the brain. Imaging studies with radiogallium scans in patients with GBM demonstrated that 67Ga localized in brain tumors.^21^^,^^22^ Recently, we demonstrated that gallium maltolate administered intravenously or orally retarded the growth of human GBM xenografts implanted orthotopically in a rat brain tumor model and significantly prolonged the survival of GBM-bearing animals.^13^^,^^23^ These studies also demonstrated that TfR1 and the iron-containing M2 subunit of RR are increased in tumor biopsies from patients with GBM and that GBM tumors in gallium-treated rats displayed an upregulation of TfR1 consistent with tumor iron deprivation.^13^

Finnegan et al first reported the synthesis of tris(3-hydroxy-4-pyranato)-gallium (gallium maltolate) as one of several water-soluble aluminum and gallium chelates that might be of medical interest.^24^ One of our authors (LRB) independently invented gallium maltolate and discovered that it could be administered orally with high gallium bioavailability.^25^ His group was the first to conduct studies on the biochemistry, pharmacokinetics, and safety of orally administered gallium maltolate in healthy human subjects and dogs.^25^ They demonstrated that single doses of as much as 500 mg/day of gallium maltolate were well tolerated with no dose-limiting or other serious toxicity, including no renal toxicity.^25^ They determined that gallium maltolate had an estimated oral bioavailability in humans of 25%-57%, much higher than the estimated oral bioavailability of gallium nitrate, at about 2%.^25^

Herein we report the results of an FDA-approved prospective single-institution Phase 1 clinical trial of oral gallium maltolate (NCT04319276) in patients with recurrent or treatment-refractory GBM. The primary objective of this trial was to determine the dose-limiting toxicity (DLT) of gallium maltolate and to provide a recommended Phase 2 dose (RP2D) for future studies. A secondary objective was to identify preliminary signals of antitumor activity with gallium maltolate within the confines of a Phase 1 study.

## Methods

The Phase 1 trial of oral gallium maltolate for the treatment of recurrent or refractory GBM was approved by the Institutional Review Board of the Medical College of Wisconsin (MCW) and conducted at the Froedtert and MCW Clinical Cancer Center. All the patients provided written consent prior to enrollment in the study. Gallium maltolate for human use was obtained from Gallixa LLC (Menlo Park, CA) by the Study Sponsor (IB/IQAI) and was compounded in gelatin capsules (500 mg/capsule) by Belmar Pharma Solutions (Golden, Colorado). The stability of the gallium maltolate capsules was monitored at the Shimadzu Laboratory for Advanced and Applied Analytic Chemistry, University of Wisconsin, Milwaukee, Wisconsin.

### Patient Inclusion Criteria

Enrolled patients were required to have had a prior histological diagnosis of GBM or molecular features of GBM, based on the 2021 WHO classification,^26^ be ≥ 18 years of age with an Eastern Cooperative Oncology Group (ECOG) performance status of 0-2, and have a diagnosis of recurrent or treatment-refractory GBM following completion of standard treatment with radiotherapy and TMZ.^1^ Patients were required to have either measurable disease per Response Assessment in Neuro-Oncology (RANO) criteria or pathologically proven recurrence.^27^^,^^28^ There was no limitation on the amount of chemotherapy or radiation patients could receive prior to enrollment in the study. Patients must have agreed to use methods for avoidance of pregnancy while on study. Acceptable laboratory parameters for eligibility were an absolute neutrophil count (ANC) of ≥ 1,500/µL, hemoglobin ≥ 9 g/dL, platelet count ≥ 100,000/µL, alanine aminotransferase (ALT) ≤ 2 x upper limits of normal (ULN), aspartate aminotransferase (AST) ≤ 2 x ULN, alkaline phosphatase ≤ 2 x ULN, total bilirubin ≤ 2 x ULN, and creatinine < 1.5 mg/dL, an estimated glomerular filtration rate of ≥ 45 as calculated by MDRD (modification of diet in renal disease), and a negative urine pregnancy test for women of childbearing age. Patients were also screened for HIV and hepatitis. Brain MRI, pulmonary function tests, and electrocardiogram were obtained within 30 days of starting gallium maltolate, and patients were required to discontinue cytotoxic chemotherapy, radiation therapy, and iron supplementation one month prior to starting gallium maltolate.

### Exclusion Criteria

Patients were excluded from the study if they had: (1) Other active malignant disease diagnosed within 12 months, apart from adequately treated non-melanoma skin cancer, adequately treated melanoma grade 2 or less, or cervical intraepithelial neoplasia. Active malignancy was considered malignancy receiving treatment. (2) Prior chemotherapy or radiotherapy within 14 days of study entry. (3) Unstable or severe concurrent medical conditions such as severe heart failure (New York Heart Association Class 3 or 4) or known lung (FEV <50%) disease; history of interstitial lung disease, history of slowly progressive dyspnea and unproductive cough, sarcoidosis, silicosis, idiopathic pulmonary fibrosis, pulmonary hypersensitivity pneumonitis, symptomatic pleural effusion, or uncontrolled diabetes mellitus. (4) Inability to tolerate oral medication. (5) Pregnant or breastfeeding. (6) Not completed all standard-of-care treatments for GBM, including surgical procedures and radiation therapy for GBM. (7) Any condition which, in the investigator’s opinion, made the patient unsuitable for study participation.

### Trial Design and Treatment

**Figure 1.**
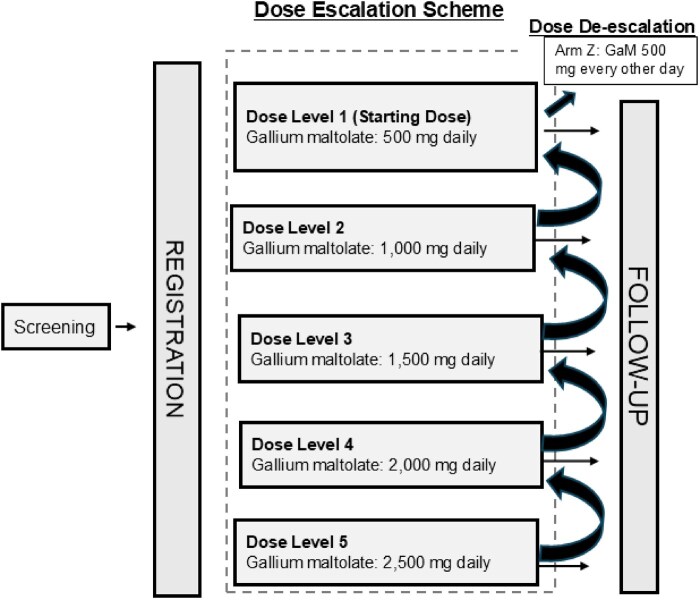
Gallium maltolate drug dose escalation schema.

An overview of gallium maltolate dose escalation plan is shown in Figure 1. A 3 + 3 Phase 1 study design was planned with the allowance that up to 6 patients could be enrolled at each level based on DLTs or the Investigator’s discretion. Accordingly, the first 3 patients received a starting dose of 500 mg of gallium maltolate daily. If DLT was not encountered at that level, a second cohort of 3 patients received 1,000 mg of gallium maltolate daily. Escalation to the next dose level was allowed only after the third patient in the previous dose level had been observed for 28 days (one treatment cycle) and no DLT was noted. If DLT was encountered in 1 of 3 patients in the dose level, 3 additional patients were treated at that dose level. If only 1 of the 6 patients experienced DLT, the next cohort of patients were advanced to the higher gallium maltolate dose level (ie 1,500 mg daily). However, if DLTs were encountered in 2 of 6 patients, subsequent patients were treated at the lower dose level, which was then considered to be the RP2D. However, the planned schedule for the first two dose levels was modified slightly. Given the limited published information regarding the adverse effects of oral daily gallium maltolate, 5 patients were treated at the first dose level of 500 mg/day, and 4 patients were treated at the second dose level of 1,000 mg/day. Since it was thought that DLTs might be encountered at the 1,500 mg/day dose level, 6 patients were treated in this cohort to allow for a planned dose-expansion group per protocol. However, because DLTs were not encountered at 1500 mg/day, approval was obtained from our Institutional Scientific Review Committee, Data Safety Monitoring Committee, and the IRB to amend the protocol to include additional dose levels of 2,000 and 2,500 mg/day of gallium maltolate.

### Patient Monitoring on Treatment

To be evaluable for treatment toxicity, all patients in the study were required to have received a minimum of one 28-day cycle of outpatient gallium maltolate without interruption. To be evaluable for signals of tumor response, patients were required to have received a minimum of 2 cycles of treatment. Tolerance to treatment was assessed by physical examinations conducted on days 1 and 15 of cycle 1 and on day 1 of subsequent cycles. Laboratory tests including complete blood count, metabolic panel, liver function panel, and serum gallium levels were obtained every two weeks for the first 2 cycles starting on the first day of cycle 1. Tumor status was assessed by brain MRI after patients had completed cycle 2 of gallium maltolate (8 weeks of treatment). Treatment with gallium maltolate was discontinued if patients had MRI evidence of GBM progression. Patients with MRI evidence of stable or responding disease and tolerance to treatment were provided the option to continue gallium maltolate until disease progression. These patients continued regular clinic visits that included laboratory testing every month and brain MRIs repeated every 2 months. Patients were removed from the study when disease progression was noted on MRI. Data from the study was collected in electronic patient clinical case report forms through the Oncore Clinical Trial Management System.

### Serum Gallium Measurements

Prior to their clinic visits, all patients had blood drawn for measurement of steady-state serum gallium concentrations on day 15 of cycle 1, on day 1 and 15 of cycle 2, and, in some patients, on day 1 of cycle 3. Each sample was analyzed for gallium content in triplicate on a single quadrupole Shimadzu ICPMS-2030 instrument equipped with a concentric nebulizer, cooled spray chamber, and plasma mini torch. Samples were injected using a Teledyne Cetac Technologies ASX-560 autosampler. The plasma was operated at RF power = 1.2 kW, plasma gas = 9.0 L/min, auxiliary gas = 1.10 L/min, and carrier gas = 0.70 L/min. The collision/reaction cell operated in helium kinetic-energy discrimination (KED) mode, with cell gas = 6.0 L min^−1^, cell voltage = −21 V, and energy filter = 7.0 V. The spray-chamber temperature was maintained at 5°C. The instrument was operated, and data were acquired using LabSolutions software (Shimadzu Corporation). External calibration for serum gallium levels was performed using multi-element standards prepared from 1,000 ppm certified stock standards (Sigma Aldrich, St. Louis, MO). The calibration standards were prepared in 2% HNO_3_ and spiked to 40 ppb indium (^115^In) as an internal standard. The calibration range spanned from 0.5 to 300 ppb (0.5, 1, 10, 25, 50, 75, 100, 150, 200, 300 ppb) and gallium levels were reported in micromolar concentrations.

For statistical analysis of serum gallium measurements, the serum Ga levels were compared between the groups using mixed-effects linear regression with fixed dose and measurement time effects and random subject effect to account for repeated measures. The outcome was log-transformed to improve normality of the residuals. Tukey’s multiple comparison adjustment was used to evaluate all pairwise comparisons. Only comparisons significant at an adjusted 5% significance level are shown.

## Results

### Patients

The reporting period for this study was from May 2022 to December 2025. The characteristics of the patients in the study are shown in Table 1; the molecular profiling of their tumors is shown in Supplementary Table S1. Fifty-seven patients were screened, and 26 consented to enter the trial. However, prior to starting gallium maltolate, one patient was unable to complete pulmonary function testing and was deemed ineligible. Another patient progressed 2 weeks after starting gallium maltolate and therefore was not evaluable for adverse effects. Of the remaining 24 patients evaluable for toxicity, 2 patients completed the first cycle of gallium maltolate but developed disease progression (detected by worsening clinical symptoms and brain MRI) in the second cycle. These patients were considered evaluable for adverse effects only. The remaining 22 patients who received 2 or more cycles of gallium maltolate were evaluable for response; they continued to be monitored for adverse effects of treatment and disease progression.

**Table 1.** Characteristics of patients with recurrent GBM

	Gallium maltolate dose (mg/day)				
	500	1,000	1,500	2,000	2,500
Number of patients	5	4	6	3	6
Median age, years (range)	61 (42-75)	46 (39-69)	59 (38-68)	52 (51-57)	69 (55-80)
Performance (ECOG*)	0-2	0-2	0-2	0-2	0-2
Race (White)	5	4	6	3	6
Gender (Male)	3	4	4	2	5
Relapse number (Patients)	1^st^ (3)	1^st^ (1)	1^st^ (6)	1^st^ (1)	1^st^ (5)
	3^rd^ (1)	2^nd^ (2)		2^nd^ (1)	2^rd^ (1)
	4^th^ (1)	3^rd^ (1)		3^rd^ (1)	
Patients with disease >1 cm^3^ with contrast enhancement	2	3	3	2	4
MGMT promoter methylated at initial diagnosis	2	0	3	2	2
IDH wt	6	4	6	3	6
Redo surgery	3	3	6	2	6

Redo surgery includes patients who had Laser Interstitial Thermal Therapy (LITT). *ECOG, Eastern Cooperative Oncology Group. All patients had IDH wild type (wt).

As shown in Table 1, patients were enrolled on study after their first or later recurrence of GBM. Sixteen patients were in their first recurrence following treatment with standard of care therapy consisting of surgery, radio-chemotherapy, and maintenance with TMZ at initial diagnosis. Eight patients had been treated for recurrent GBM prior to enrollment in the study; prior treatment details of these patients are shown in Supplementary Table S2.

*Adverse effects:* All 24 patients who completed at least one treatment cycle tolerated oral gallium maltolate well. The adverse effects experienced by patients were reported as grade 1 or 2. There were no grade 3 or higher adverse effects noted. The adverse effects of treatment at each dose level are shown in Table 2. The predominant side effects experienced by patients were gastrointestinal (diarrhea, fatigue, and nausea). Patients on the highest dose of gallium maltolate who developed diarrhea required intervention with dietary modification and loperamide.

**Table 2.** Number of patients experiencing adverse effects

Adverse Effects	Grade 1	Grade 2				
GaM dose, mg/d	All doses	500	1000	1500	2000	2500
Number of patients (percent)	*n* = 24	*n* = 5	*n* = 4	*n* = 6	*n* = 3	*n* = 6
Diarrhea	12 (50)	0	0	1 (16)	0	2 (33)
Fatigue	6 (25)	0	0	2 (17)	0	0
Nausea	5 (21)	1 (20)	0	0	0	0
Anorexia	3 (12)	0	0	0	0	1 (16)
Muscle cramps	3 (12)	1 (20)	0	0	0	0
Constipation	2 (9)	1 (20)	0	0	0	0
Dysgeusia	2 (9)	0	0	0	0	0
Dyspepsia	2 (9)	0	0	0	0	0
Tongue soreness	1 (4)	1 (20)	0	0	0	0
Anemia	1 (4)	0	0	0	0	0
Arthralgia	1 (4)	0	0	0	0	0
Confusion	1 (4)	0	0	0	0	0
Cough	0	0	0	1 (16)	0	0
Dizziness	1 (4)	0	0	0	0	0
Fecal incontinence	1 (4)	0	0	0	0	0
Weight gain	1 (4)	0	0	0	0	0
Weight loss	1 (4)	0	0	0	0	0
Headache	1 (4)	0	0	1 (16)	0	0
Paresthesia	1 (4)	0	0	0	0	0

No grade 3 or higher adverse effects were encountered.

Regardless of the dose of gallium maltolate administered, in all patients the serum levels of calcium, magnesium, creatinine, estimated glomerular filtration rate, blood urea nitrogen, potassium, uric acid, phosphorus, sodium, potassium, and bicarbonate remained within normal limits during treatment. Fifteen patients had minor sporadic elevations in serum chloride ranging from 106 to 114 mEq/L (ULN 105 mEq/L) during treatment, most likely secondary to diarrhea.

Patients were eligible for enrollment in the study if they had ALT, AST, total bilirubin, and alkaline phosphatase levels of ≤ 2 x ULN prior to starting treatment with gallium maltolate. Patient C-09 treated with 1,000 mg/day gallium maltolate developed a 3.0-fold elevation in ALT above the ULN at the end of his second cycle at which time he was found to have disease progression on MRI at this time, further treatment with gallium maltolate was discontinued. Patient C-19 treated with 2,000 mg/day developed a 2.2- and 2.1-fold elevation in ALT and AST above the ULN, respectively, on day 15 of cycle 2; these levels decreased to a 1.7-fold elevation with continuation of gallium maltolate during cycle 3. At the end of cycle 3, treatment was discontinued due to disease progression; at this point her ALT was 2.5 above the ULN. None of the other patients in the study developed elevations in AST, ALT. bilirubin, or alkaline phosphatase greater than a 2-fold increase above the ULN during treatment, including 6 patients treated with the higher dose of 2,500 mg/day of gallium maltolate.

Another side effect of gallium maltolate in patients was a downward trend in hemoglobin and RBC mean corpuscular volume (MCV) relative to pretreatment levels. One patient developed grade 1 anemia with a decrease in hemoglobin to <10 g/dL (Table 2). Nine patients had a decrease in MCV below the normal low value of 80 fL with accompanying decreases in their hemoglobin relative to pretreatment values in eight patients (Table 3). In this group, patient C-05 had a history of iron-deficiency anemia with a decreased MCV and normal hemoglobin before starting gallium maltolate. In 8 of the 9 patients, MCV values returned to pretreatment levels by 15 weeks following cessation of gallium maltolate, thus indicating a cause-and-effect relationship between gallium and the development of red cell microcytosis. Patient C-15 who has been on gallium maltolate continuously for 33 cycles had a decrease in MCV, which has remained unchanged with ongoing treatment (Table 3). Although most of the other patients experienced a treatment-related decrease in MCV, none of these patients had a decrease in MCV below normal. Importantly, none of the patients were symptomatic from changes in MCV or hemoglobin and did not require clinical intervention with red blood cell transfusions. Neutrophil counts were not decreased by gallium maltolate regardless of the dose level. A trend toward an increase in mean platelet counts from baseline was noted in some patients at the highest dose of gallium maltolate, however, these values remained within normal limits.

**Table 3.** Patients with decreases in RBC MCV below normal during treatment

Patient ID	Cycles	Dose	MCV-1	MCV-2	Hgb-1	Hgb-2
C-01	8	500	92	78	11.5	12.4
C-04	6	500	92	78	16.2	15.3
C-05	25	500	74	51	12.5	10.3
C-06	10	500	96	70	10.7	10.4
C-14	6	1,500	86	67	15.6	14.2
C-15	32	1,500	88	62	15.9	11.3
C-19	4	2,000	92	78	13.2	11.1
C-23	4	2,500	95	69	13.2	9.2
C-25	3	2,500	88	73	13.7	12.4

Hemoglobin **(**Hgb) -1 and -2, and mean corpuscular volume (MCV)-1 and -2 reflect the values before and during treatment with gallium maltolate, respectively. The lower limit of normal for MCV is 80 fL.

### PFS and OS

**Figure 2.**
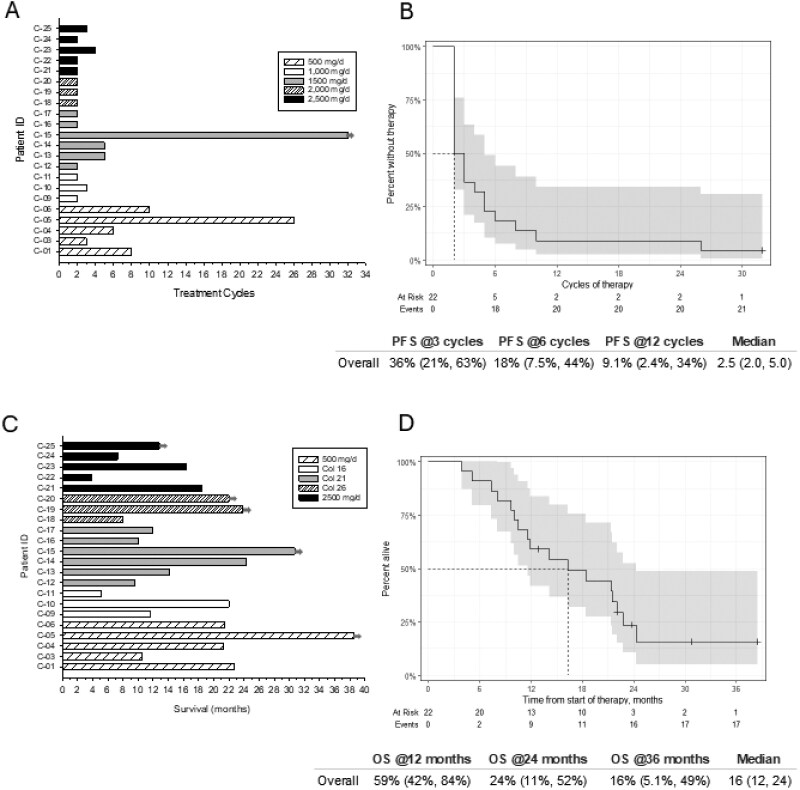
PFS and OS of patients treated with oral gallium maltolate. Twenty-two patients were evaluable for treatment outcomes. **(A)** The number of cycles of gallium maltolate received by each patient is shown on the x-axis. Since treatment was discontinued when disease progression was detected on MRI, the length of the bar represents both the treatment duration and PFS. Patient C-15 experienced a partial response and remains on treatment (arrow). (**B)** Corresponding Kaplan-Meier analysis for PFS. (**C)** Patient OS was measured from the start of treatment with gallium maltolate. Five living patients are identified by arrows, while the other patients are deceased. (**D)** Corresponding Kaplan-Meier analysis for OS.

A secondary objective of the study was to look for, within the context of a Phase 1 trial, signals suggestive of tumor response to treatment based on RANO criteria. Two of the 24 patients enrolled on trial were not included in this analysis because these patients did not complete the required minimum of 2 cycles (8 weeks) of treatment to be evaluable for analysis of PFS and OS. For the 22 eligible patients, the duration of treatment was measured from the start to the cessation of gallium maltolate. Since patients received treatment continuously until progression was detected on brain MRI, the duration of treatment was equivalent to their PFS (Figure 2A). Here, 11 patients showed tumor progression on MRI after 2 cycles and discontinued gallium maltolate. The remaining 11 patients had stable disease on RANO criteria and continued treatment until progression was documented on MRI, at which point gallium maltolate was discontinued. As shown in the swimmer’s plot in Figure 2A, an additional 3 patients (C-01, C-06, and C-05) progressed after 8, 10, and 26 cycles, respectively. Patient C-15 experienced a partial response (>50% reduction in tumor size) and remains on treatment for 33 cycles of gallium maltolate (Figure 2A). Patient OS is shown in the swimmer’s plot in Figure 2C. Five of 22 patients who received at least 2 cycles of gallium maltolate are alive, 4 of whom are beyond 18 months with 2 of them at 31 and 39 months since starting treatment with gallium maltolate. The corresponding Kaplan-Meier analyses for PFS and OS are shown in Figure 2B and D, respectively.

### Gallium Pharmacokinetics

**Figure 3.**
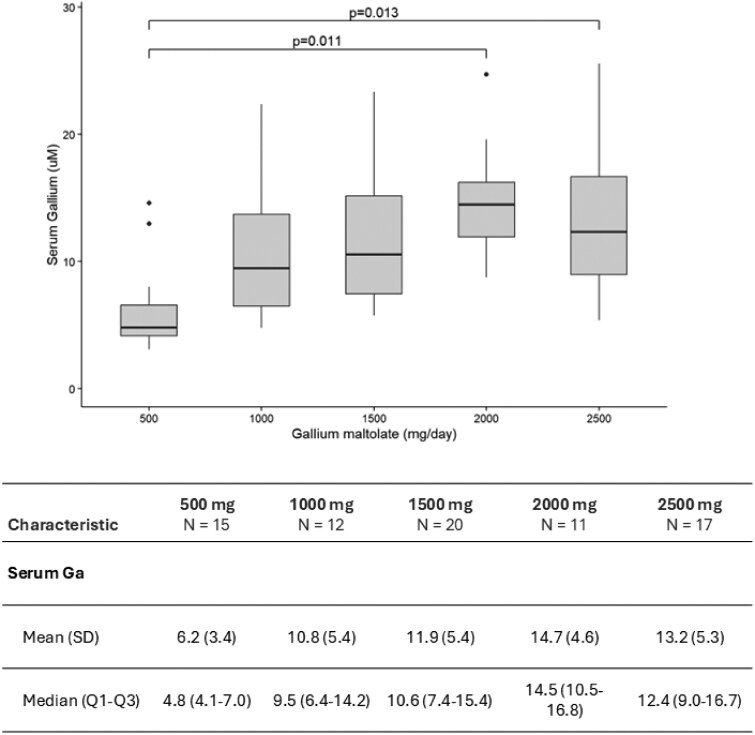
Steady-state serum gallium levels during the first 2 cycles of treatment. Gallium levels were measured on cycle 1 day 15, cycle 2 days 1 and 15, and following the completion of cycle 2. N refers to the number of serum samples analyzed for gallium levels at each dose. See text for details of statistical analysis.

Figure 3 shows significantly higher serum gallium levels, obtained during the first 2 cycles of treatment, for doses higher than 500 mg/day compared to the 500 mg/day dose. The mean serum gallium levels corresponding to the gallium maltolate doses of 500 mg/day, 1,000 mg/day, 1,500 mg/day, 2,000 mg/day, and 2,500 mg/day were 6.21 µM, 10.78 µM, 12.21 µM, 14.80 µM, and 14.33 µM, respectively.

### Determination of RP2D

Whereas conventional Phase 1 trials involve progressive increases in the dose of a drug under study to the point that patients encounter grade 3-4 toxicities, we incorporated both gallium pharmacokinetics and clinical symptoms into our consideration of the RP2D for oral gallium maltolate. First, the finding that serum gallium levels plateaued at gallium maltolate doses of 2,000-2,500 mg/day in 9 patients indicated that an increase in the dose beyond 2,500 mg/day would not produce a further elevation in serum gallium. Second, consistent with earlier reports that diarrhea was the DLT noted with continuous infusion gallium nitrate,^29^ our Phase 1 trial revealed that grade 2 diarrhea requiring intervention was encountered in one-third of the patients treated with 2,500 mg/day; this strongly suggested that further elevations in dose level would only increase this toxicity. Thus, it was determined that the appropriate RP2D for oral gallium maltolate should be 2,000 mg/day.

## Discussion

The primary objective of our Phase 1 clinical trial was to determine the safety of oral gallium maltolate administered daily at increasing dose levels in patients with recurrent GBM and to establish an RP2D for gallium maltolate for future trials. Our results advance the observations made in a prior dose-escalation Phase 1 trial of 24 patients treated with multiple cycles of gallium maltolate at doses of 50, 150, or 500 mg per day on a schedule of 28 days on followed by 14 days off treatment.^30^ That study, which was reported in abstract form at the EORTC-NCI-AACR symposium in 2002, was conducted in patients with advanced prostate cancer, bladder cancer, myeloma, or lymphoma. The investigators concluded that 500 mg per day of oral gallium maltolate was safe and planned to continue their trial with higher doses.^30^ The continuation of that clinical trial found oral gallium maltolate to be well tolerated with no reported DLT at doses as high as 3,500 mg administered at the dosing schedule of 28 days on and 14 days off treatment.^31^^,^^32^ Because the subjects in our Phase 1 dose-escalation trial represented a patient population not previously studied and treated with a different dosing schedule, we chose to initiate the trial with 500 mg gallium maltolate administered daily without interruption.

Oral gallium maltolate was well tolerated by patients with only grade 1 or grade 2 toxicities encountered. However, at the dose of 2,500/day of gallium maltolate, one-third of patients required therapeutic intervention for diarrhea. Based on this clinical finding and the plateau in serum gallium levels, we conclude that the DLT for daily gallium maltolate is diarrhea and that the RP2D for future studies should be 2,000 mg/day, one dose level lower than the DLT. It is relevant to note that Warrell et al also reported diarrhea to be the DLT in patients with relapsed lymphoma treated with a 7-day continuous intravenous infusion of gallium nitrate.^29^

Unlike the experience with intravenous gallium nitrate, none of the patients in the present study developed renal dysfunction with daily oral gallium maltolate. Renal function remained normal in patients who received treatment. Thus, the lack of nephrotoxicity with gallium maltolate contrasts strikingly with gallium nitrate, where a dose-dependent increase in creatinine was encountered in patients and required dose reduction with concurrent hydration to reduce the risk of renal damage.^33^  These differences are best explained by considering the aqueous chemistry of gallium.^34^^,^^35^ When gallium nitrate solution (which contains sodium citrate) is directly introduced into the bloodstream, much of it will likely be present as the gallate ion, [Ga(OH)4]^-^.^14^ As a small, charged ion, gallate will be rapidly excreted by the kidneys, where it can reach concentrations high enough to react with phosphate in the renal tubules to precipitate as gallium calcium phosphate.^35^ In contrast, gallium from orally ingested gallium maltolate binds predominantly to Tf in the circulation to form stable Tf-Ga, which can target TfR1-bearing cells.^9^^,^^10^^,^^15^^,^^36^^,^^37^

While none of the patients in our study developed symptomatic anemia requiring RBC transfusions, a decrease in MCV accompanied by a decline in hemoglobin below pretreatment levels was noted in some patients treated with oral gallium maltolate; this was not unexpected. The basic mechanisms underlying gallium-related anemia were investigated in murine erythroleukemia cells, which can be induced by dimethyl sulfoxide to synthesize hemoglobin. Tf-Ga was shown to inhibit hemoglobin synthesis in these cells by decreasing the incorporation of iron into heme. Gallium inhibition of hemoglobin synthesis could be restored by iron-pyridoxal isonicotinoyl hydrazone and Tf-iron, thus indicating that the anemia in patients receiving gallium maltolate was not due to myelosuppression but was related to gallium’s interference with Tf-iron transport and the utilization of iron for hemoglobin synthesis by the erythron.^38^ Consistent with this mechanism, Warrell et al reported microcytic anemia in patients treated with continuous infusion gallium nitrate,^29^ and Seligman et al later demonstrated that free erythrocyte zinc protoporphyrin, a marker of early cellular iron deficiency, was increased in RBCs obtained from patients during treatment with gallium nitrate for metastatic bladder cancer.^39^ In the context of toxicity, the development of reversible mild to moderate RBC microcytosis with gallium maltolate in our study is a relatively mild adverse effect. Importantly, RBC microcytosis should also be considered evidence that oral gallium maltolate interferes with cellular iron homeostasis in patients. The effects of long-term treatment with gallium maltolate on erythropoiesis are best exemplified by patient C-15 who had been on treatment for 32 cycles. This patient developed a low MCV early during his treatment and remains on gallium maltolate without further worsening of his mild anemia and microcytosis over time.

Evidence for dose-responsive gastrointestinal absorption of oral gallium maltolate was provided by measurements of steady-state gallium levels in the blood, which demonstrated a significant increase in serum gallium levels between 500 mg/day and the higher doses of 2,000 and 2,500 mg/day. The pattern of serum gallium levels at the latter doses raises the possibility that the gastrointestinal absorption of oral gallium maltolate involves a saturable transport system in enterocytes and that blood levels of gallium will likely begin to plateau at doses greater than 2,000 mg administered once a day. Also, although patients were instructed to take the drug on an empty stomach 2 hours outside of a meal, it is possible that some patients were not fully compliant with this guideline and may have taken gallium maltolate close to or with meals. Comparisons can be made between the absorption of oral iron and gallium, as both metals bind Tf in the blood.^9^ The steps involved in the gastrointestinal absorption of iron are highly regulated to ensure that appropriate amounts of iron enter the blood. The amount of iron absorbed by healthy adults is limited physiologically and does not increase linearly when the amount of iron ingested is increased.^40^ In contrast to iron, little is understood regarding the steps involved in regulating gallium absorption from the gut. Further research is needed to better understand this process and to determine whether another dosing schedule such as twice-daily drug administration may yield higher gallium blood levels.

The secondary objective of our trial was to look for signals of tumor response to treatment. Kaplan-Meier analysis showed a median PFS of 2.5 months and a median OS of 16 months for patients treated with gallium maltolate. The latter compares favorably with the median OS of 5.5 to 12.6 months reported in a meta-analysis of 42 trials involving 5,236 patients treated for recurrent GBM with various interventions including chemotherapy, re-operation, re-irradiation, and novel therapies alone and in combination.^4^ Conclusions regarding the efficacy of gallium maltolate in GBM cannot be made based on the results of our study, as this will require a statistically powered, prognostic marker-stratified Phase 2 trial with the RP2D of this drug in the future.

Our preclinical research led us to propose a mechanistic model in which gallium’s activity against GBM, as in other cancers, involves its ability to highjack the delivery and utilization of iron for aggressive tumor growth.^14^^,^^15^^,^^41^^,^^42^ In this model, orally ingested gallium enters the circulation and binds to Tf, akin to iron. Tf-Ga then enters the brain via Tf receptors on the luminal surface of vascular endothelial cells of the BBB that normally deliver iron to the brain.^43^ Within the brain, the choroid plexus secretes Tf, which can serve to bind gallium and deliver it to TfR1 present in high densities on GBM cells.^44^ Gallium uptake by GBM may also be facilitated by its binding to Tf secreted by GBM cells as an autocrine factor to enhance its acquisition of iron.^12^ Preferential homing of Tf-Ga to TfR1 on GBM cells is possible because normal glial cells surrounding GBM generally do not express detectable levels of TfR1.^44^

## Conclusions

Our study is the first Phase 1 clinical trial to fully evaluate a gallium compound in GBM and to establish the safety and tolerability of daily oral gallium maltolate in this setting. We have determined 2,000 mg/day to be the RP2D for oral gallium maltolate based on the plateau in serum gallium levels coupled with the adverse effects of diarrhea encountered at higher levels. Further studies of gallium maltolate in GBM are warranted.

## Supplementary Material

vdag154_Supplementary_Data

## Data Availability

This is an FDA-approved Phase 1 clinical trial. The data from this study is being reported to the FDA and will be made available to readers through ClinicalTrials.gov.
