# Supplementary material for: A phase 1 trial of iron metabolism-targeting oral gallium maltolate in recurrent and refractory glioblastoma
Source: Neurooncol Adv. 2026 Jun 8;8(1):vdag154. doi: 10.1093/noajnl/vdag154 (PMC13283452; doi:10.1093/noajnl/vdag154)
Supplement: vdag154_Supplementary_Data [file vdag154_supplementary_data.docx]

**S1. Supplemental to Table 1**

**Molecular characteristics of patients treated with gallium maltolate**

| Patient | IDH | MGMT | TERT | PTEN | EGFRvIII | EGFR | Gain of 7 | Loss of 10 | CDKN2A/B |
| --- | --- | --- | --- | --- | --- | --- | --- | --- | --- |
| C-01 | wt | uM | Mutated | Loss | Not Mut | Not Amp | Absent | Absent | Loss |
| C-03 | wt | uM | Mutated | Loss | Not Mut | Not Amp | Absent | Absent | Loss |
| C-04 | wt | uM |  |  |  |  |  |  |  |
| C-05 | wt | M | Mutated | No Loss | Not Mut | Amp | Absent | Absent | Loss |
| C-06 | wt | M |  |  |  |  |  |  |  |
| C-08 | wt | uM | Mutated |  |  |  |  |  |  |
| C-09 | wt | uM | Mutated | Loss | Not Mut | Not Amp | Absent | Absent | Loss |
| C-10 | wt | uM |  |  |  |  |  |  |  |
| C-11 | wt | uM |  |  |  |  |  |  |  |
| C-12 | wt | M | Mutated | Loss | Mutated | Amp | Absent | Absent | Loss |
| C-13 | wt | M | Mutated | No Loss | Not Mut | Not Amp | Present | Present | No loss |
| C-14 | wt | uM | Mutated | No Loss | Not Mut | Not Amp | Absent | Absent | No loss |
| C-15 | wt | M |  |  |  |  |  |  | Loss |
| C-16 | wt | uM |  |  |  |  |  |  |  |
| C-17 | wt | uM | Mutated | Loss | Not Mut | Not Amp | Absent | Absent | Loss |
| C-18 | wt | uM |  |  |  |  |  |  |  |
| C-19 | wt | M | Mutated | Loss | Mutated | Amp | Absent | Absent | Loss |
| C-20 | wt | M |  |  |  |  |  |  |  |
| C-21 | wt | uM | Mutated |  |  |  |  |  |  |
| C-22 | wt | M |  |  |  |  |  |  |  |
| C-23 | wt | M |  |  |  |  |  |  |  |
| C-24 | wt | uM | Mutated | No Loss | Not Mut | Not Amp | Present | Present | No loss |
| C-25 | wt | uM |  | Loss |  |  |  |  |  |
| C-26 | wt | uM |  |  |  |  |  |  |  |

Legend: IDH wt, wild type; MGMT uM, unmutated, M, mutated; Amp, amplified. Mut, mutated.

**S2. Supplemental to Table 1**

**Prior treatments for recurrent GBM received by patients**

| Patient | Recurrence | Treatment |
| --- | --- | --- |
| C-01 | 1^st^  2^nd^  3^rd^ | Redo surgery, RT/TMZ followed by TMZ (12 cycles)  TMZ for 12 cycles  Surgery followed by re-irradiation + bevacizumab |
| C-03 | 1^st^  2^nd^ | Redo surgery followed by re-irradiation  Redo surgery (second recurrence) followed by TMZ (5 cycles) |
| C-08 | 1^st^  2^nd^ | Redo surgery followed by CCNU (2 cycles)  Redo surgery |
| C-09 | 1^st^  2^nd^ | Redo surgery followed by re-irradiation  Redo surgery followed by lomustine (5 cycles) |
| C-10 | 1^st^  2^nd^ | Redo surgery with glide wafer placement followed by 12 cycles of TMZ  Redo surgery |
| C-18 | 1^st^ | Redo surgery followed by lomustine |
| C-20 | 1^st^  2^nd^  3^rd^ | Redo surgery followed by lomustine (6 cycles)  Redo surgery followed by lomustine (6 cycles)  Redo surgery |
| C-22 | 1^st^  2^nd^ | TMZ and TTFields  Redo surgery |

**Legend:** Eight of twenty-four patients received treatment for recurrent GBM prior to enrollment in the Phase 1 trial.
